# Applications of Machine Learning in Automotive Engineering: Editorial to the Special Issue

**DOI:** 10.3390/s26196283

**Published:** 2026-10-03

**Authors:** Hwan-Sik Yoon

**Affiliations:** Department of Mechanical Engineering, The University of Alabama, P.O. Box 870276, Tuscaloosa, AL 35487-0276, USA; hyoon@eng.ua.edu

## 1. Introduction

The automotive industry is currently undergoing one of the most significant technological transformations in its history. The rise of automated and connected vehicles [1], together with the growing market share of electric vehicles and their continually improving performance [2], is reshaping how vehicles are designed, controlled, monitored, and maintained. These transformations present considerable engineering challenges as well as new opportunities, and machine learning (ML) and artificial intelligence (AI) have emerged as key enabling technologies for addressing them, spanning the design and control of vehicles to their monitoring and maintenance.

Over roughly the past decade, ML methods, from classical statistical learning to deep neural networks and reinforcement learning, have moved from academic curiosity to practical deployment across nearly every subsystem of the modern vehicle, a shift documented in a growing body of review literature on machine learning and artificial intelligence in automotive engineering [3,4,5]. Much of this activity is organized around the vehicle’s principal data sources, each the subject of a substantial and still-growing survey literature in its own right: cameras [6], LiDAR [7], radar [8], and in-vehicle networks such as the Controller Area Network (CAN) bus [9]. These sensing and communication channels are now routinely processed using learned models to perceive the environment, estimate vehicle and traffic states, plan and execute safe maneuvers, detect anomalies and cyberattacks, forecast battery behavior, and monitor the driver. Because these functions are so heterogeneous, progress is often reported within disciplinary silos including computer vision, control theory, cybersecurity, and human factors, among others. This Special Issue, “Applications of Machine Learning in Automotive Engineering,” was launched to bring representative examples of this work together in one venue and to give readers a cross-section of where machine learning is currently making a difference in automotive engineering.

The call for papers invited contributions on machine learning and its application in automotive systems; modeling, simulation, and control of automotive systems inspired by machine learning or AI; advanced sensing and actuation via machine learning; controls based on reinforcement learning; connected and automated vehicles; predictive maintenance; advanced driver assistance systems (ADAS); and human–machine interfaces. The response was broad. The fifteen accepted articles, summarized below, address problems across perception, state and trajectory estimation, control, vehicle health and security, and driver monitoring, drawing on data sources as varied as LiDAR point clouds, monocular camera video, in-vehicle CAN-bus traffic, GPS logs, and battery cell voltages.

## 2. Overview of the Contributions

For clarity, the fifteen contributions to this Special Issue are grouped below into five thematic clusters: perception and environmental sensing, vehicle state and trajectory estimation, learning-based control and decision-making, vehicle health, energy, and cybersecurity, and driver monitoring and human–machine interaction. However, several papers naturally address more than one thematic area.

### 2.1. Perception and Environmental Sensing

The first cluster of papers addresses how vehicles and roadside infrastructure sense their surroundings. Wu and Wei (Contribution 1) propose GENet, a geometry-enhanced network for real-time LiDAR semantic segmentation that avoids the spatial information loss typical of 2D-projection-based methods while maintaining the inference speed required for autonomous driving and mobile robotics. Working with roadside cameras rather than onboard sensors, Li and Yoon (Contribution 2) improve the calibration of traffic surveillance cameras by combining a genetic algorithm with particle swarm optimization, and, in a related Editor’s Choice article, extend this camera-based approach to localize vehicles in three-dimensional world coordinates at a signalized intersection using a single camera (Contribution 3), offering a low-cost alternative to LiDAR- or multi-camera-based localization for traffic management. Reveles-Gómez et al. (Contribution 4) target a safety-critical perception task closer to the vehicle itself, using multimodal convolutional neural networks to detect pedestrians in reverse-camera footage, a scenario in which limited field of view and close range make detection especially difficult. Completing this cluster, Nadeem et al. (Contribution 5) apply deep learning to road feature detection for ADAS, identifying lane markings, signage, and other roadway elements that support downstream driving assistance functions. Together, these five articles illustrate how learned perception, whether onboard or infrastructure-based, continues to be refined for accuracy, robustness, and real-time performance.

### 2.2. Vehicle State and Trajectory Estimation

A second cluster focuses on estimating where a vehicle is, how it is moving, and how it is likely to move next. Kim et al. (Contribution 6) present a delay-compensated, lane-coordinate vehicle state estimator that fuses a camera, an inertial measurement unit, and steering-angle sensing, all low-cost, to support ADAS and autonomous-driving functions that depend on accurate lane-relative positioning. Lee et al. (Contribution 7) address a related but distinct problem: rather than estimating vehicle state, they classify whether an ADAS is actually active during real-world driving, using synchronized CAN-bus and smartphone IMU data, with applications in safety monitoring and post-accident forensics. Looking further ahead in time, Zhang (Contribution 8) investigates domain generalization in trajectory prediction using the rounD roundabout dataset, combining gated recurrent units with graph-based interaction modules to improve how well trajectory-prediction models transfer across different roundabout geometries and traffic conditions, which is a practical concern for models trained at one location and deployed at another.

### 2.3. Learning-Based Control and Decision-Making

Two contributions focus on using learned policies to make driving decisions. Zhao et al. (Contribution 9) propose a safe reinforcement learning framework for autonomous highway driving that explicitly incorporates constraints to reduce collision risk, including in long-tail scenarios that are underrepresented in training data, and report improved safety and stability relative to unconstrained reinforcement learning baselines. Kim et al. (Contribution 10) apply deep reinforcement learning to lane-keeping control, conditioning the policy on the behavior of surrounding vehicles so that the controller can adapt to nearby traffic rather than relying on lane geometry alone. Both articles reflect a broader trend in the field in which reinforcement learning is increasingly evaluated not only on nominal performance but on its robustness and safety under realistic, rare, or adversarial driving conditions.

### 2.4. Vehicle Health, Energy, and Cybersecurity

A fourth group of papers looks inward, at the health, energy, and security of the vehicle itself. Two articles address the growing importance of protecting the in-vehicle network. Kim et al. (Contribution 11) propose an adaptive, single-threshold, autoencoder-based intrusion detection system for CANs that achieves high detection accuracy while reducing the computational overhead required for in-vehicle deployment, and Dini and Saponara (Contribution 12) design and experimentally assess real-time anomaly detection techniques for automotive cybersecurity more broadly, evaluating their approach under realistic attack scenarios. On the energy side, Pourbunthidkul et al. (Contribution 13) develop a cascaded, two-stage LSTM framework for multi-cell voltage forecasting and state-of-charge estimation in electric vehicle batteries, with particular attention to the performance degradation that battery management systems face in high-temperature climates. Rivera-Campoverde et al. (Contribution 14) take a complementary, environmental angle, combining GPS logging with machine learning to estimate real-driving emissions from sedans, SUVs, and pickup trucks, offering a practical, low-cost alternative to laboratory emissions testing.

### 2.5. Driver Monitoring and Human–Machine Interaction

Finally, Pyeon et al. (Contribution 15) address the human side of the human–machine system, proposing a deep-learning-based system that predicts whether a driver’s hands are on or off the steering wheel using in-vehicle data. As partial automation becomes more common, reliably detecting hands-on/hands-off status is an important safeguard for systems that still require timely driver takeover.

## 3. Conclusions and Future Outlook

The fifteen articles in this Special Issue show how thoroughly machine learning has been woven into automotive engineering practice—not as a single headline application such as end-to-end autonomous driving, but as a toolkit applied to a wide range of narrower, safety- and efficiency-relevant problems. Several cross-cutting themes emerge. First, sensor fusion and low-cost sensing remain a recurring concern: several contributions (Contributions 2,3,6,14) deliberately favor cameras, GPS, and other inexpensive sensors over LiDAR or specialized instrumentation, using machine learning to extract more information from cheaper hardware. Second, real-time performance is treated as a first-class design requirement rather than an afterthought, from LiDAR segmentation (Contribution 1) to intrusion detection (Contribution 11), reflecting the constraints of embedded and in-vehicle deployment. Third, robustness under realistic and rare conditions such as long-tail safety scenarios for reinforcement learning (Contribution 9), cross-location generalization for trajectory prediction (Contribution 8), and high-temperature operation for battery models (Contribution 13), is increasingly treated as important as average-case accuracy. Fourth, cybersecurity and vehicle-health monitoring (Contributions 4,11,12) are now firmly part of the automotive machine learning agenda, reflecting the reality that connected vehicles are also networked, and therefore attackable, systems, a concern that mirrors trends in the broader literature on learning-based CAN intrusion detection [10].

Based on the contributions in this collection, we anticipate continued research in several directions. These include tighter integration of perception, state estimation, and control into unified learning pipelines, more systematic evaluation of the safety and robustness of learning-based controllers before real-world deployment [11], broader use of low-cost and infrastructure-based sensing to complement onboard sensor suites, and greater attention to the driver as an active participant in increasingly automated systems rather than solely as a fallback. We also note that predictive maintenance, although explicitly included in the original scope of this Special Issue, attracted comparatively few submissions relative to perception and control. As vehicle health prognostics continues to mature as an active research area [12,13], we encourage future research that more closely connects it with the perception, state estimation, and control themes represented in this collection. We hope this Special Issue provides a useful snapshot of the current state of the field and serves as a starting point for researchers pursuing these directions.
